# Dynamic analysis of lathe bed of woodworking CNC machining center based on the modeling of joint surface

**DOI:** 10.1371/journal.pone.0277919

**Published:** 2022-11-28

**Authors:** Yin-Kun Sun, Jun Hua, Yan-Na Li, Guang-Wei Chen, Ming-Hui Liu

**Affiliations:** 1 College of Mechanical and Electrical Engineering in Northeast Forestry University, Harbin, Heilongjiang, China; 2 Harbin Research Institute of Forestry Machinery, the State Forestry Administration, Harbin, Heilongjiang, China; University of Vigo, SPAIN

## Abstract

Based on the finite element theory, a joint-plane modeling method is employed to connect the corresponding nodes at the joint surface of the woodworking computer numerical control (CNC) machining center bed with a 2-node 12-degree-of-freedom unit. A spatial element model is established, which can show the state of the nodes between joint surfaces when they are stretched, compressed, or twisted; and it can help build a woodworking CNC machining center on a finite element model of bed with the characteristics of the joint surface. The simulated analysis is performed on the model and is compared with the result of simulated analysis on the bed model that ignores the characteristics of the joint surface and modal experiment. The comparison verifies the effectiveness of the modeling method based on the characteristics of the joint surface. The weak link of the machine bed structure is analyzed and optimized. The natural frequency of the bed is improved by2.55% ~ 11.3%. The displacement is reduced by a maximum of 19.4%, and dynamic performance of the bed is improved.

## 1. Introduction

Modern industry can provide a highly flexible, individualized, and digital production and service. With the standardized protocol, interoperability between networked machines is realized and dynamic reorganization of machinery and modules in the factory is achieved [1]. Woodworking, the same as other industries, is entering the era of Industry 4.0, conducting digital production and processing. To meet the personalized needs of the product, the woodworking deeply integrates information technology with manufacturing technology, forming a wood-processing intelligent system based on the industrial Internet of Things (IoT). Therefore, intelligent manufacturing is an inevitable trend in development of woodworking machinery. The processing technology and production function are continuedly improved alongside with profound integration of information and manufacturing technologies, giving higher demand for dynamic performance of woodworking machinery to ensure maximum productivity, output, and reliability of the overall manufacturing process [2]. Woodworking CNC machining center, as the core equipment in IoT of the digital wood production and processing, can connect other mechanical equipment to achieve an equilibrium quantity and optimize cost in the possibly shortest time to efficiently produce highly personalized products. Its dynamic performance influences the efficiency and reliability of the entire intelligent production system.

In the mechanical structure, the surface in which the parts and components contact each other is a mechanical joint surface. Performance of the joint surface can affect the dynamic characteristic of mechanical structure. 30%–50% of the machine tool stiffness depends on the stiffness of the joint surface; more than 90% of the machine tool damping value is derived from the damping of the joint surface; and 60% of the machine vibration originates from the characteristics of the joint surface [3]. Characteristics of the joint surface can be affected by many factors, most of which are nonlinear factors. Various processing methods and application conditions are intertwined, resulting in very complex characteristics of the interface. For the complexity, establishing a theoretical model that accurately characterizes the stiffness and damping of the joint surface is the key to establishing a finite element model of the mechanical structure, and it is the premise for predicting and analyzing the dynamic characteristics of the mechanical structure.

Some specialists and academics have conducted extensive research on the subject of the dynamic characteristics of machine tools. Hao Y. P. et al. proposed a multi-source signal fusion flutter detection approach based on WPD and power entropy, which can more accurately detect the existence of early flutter and predict its severity [4]. Zhu L. D. et al. analyzed the research progress of milling flutter prediction, detection, and suppression, and the research proposed that integrating flutter prediction, detection, and suppression units into intelligent machine tools or intelligent spindles is the future path of research [5]. In addition to studying the main vibration characteristics of machine tool structure under cutting conditions, Huang Q. et al. proposed a method to predict the modal response signal and identify the dominant vibration mode of machine tools [6]. The aforementioned research results indicate that the dynamic characteristics of machine tools have a significant effect on the precision and chatter of the machining system, which must be studied further to improve the quality and efficiency of machining.

Some experts and scholars mainly focus on the experimental research and analysis of the dynamic characteristics of the whole machine and various mechanical system structures of woodworking machinery. Pimenov D. Y. et al. [7] studied the dynamic characteristics of woodworking CNC machining center and analyzed the amplitude-frequency characteristic of the CNC machining center and the dynamic performance of the woodworking CNC machining center during external excitation and milling. Noda [8] used the bending theory to derive the equation of motion under the action of thermal load and plastic strain of the tensioned circular saw. To study the influence of tension conditions on the dynamic characteristic of tensioned circular saws and find out the appropriate tension condition; Marinenov [9, 10] analyzed the deformation of the large band saw machine spindle and transmission system caused by the static and dynamic load; and parametric equations reflecting the deformation of each system are obtained to ensure safe and reliable operation of the machine related to various optimizations. The current research on dynamic characteristic and vibration of woodworking machinery generally ignores the influence of the characteristics of the joint surface, and few studies can combine theoretical analysis with experimental methods.

In the process of optimizing the machine tool structure, Shen L. et al. performed sensitivity analyses on each component based on the results of modal tests, chose the optimization target, and finished optimizing the spindle box, pillar, and lathe bed [11]. Based on the natural development law of leaf veins, Hao Y. P. et al. proposed a new method to design the architecture of reinforcement inside the supporting sections of machine tools [12]. The optimization objectives and design variables of the biomimetic CNC turntable were chosen by Lui S. H. et al. based on the results of thermodynamic coupling and modal analysis. They then used the sensitivity analysis method to investigate the influence law of the design variables on the optimization objectives, found the biomimetic CNC turntable’s optimal solution through response surface optimization, and completed the optimization design [13]. The aforementioned research results demonstrate that the current mechanical structure optimization design primarily uses mathematical programming as its core, is based on a number of research methodologies, and increases optimization accuracy while maximizing a greater number of objectives.

This paper offers a novel mechanical structure modeling method based on finite element theory that takes into account the dynamic characteristics of the joint surface. In this method, the mechanical structures of each part are combined and connected one-to-one corresponding nodes on the joint surface using two-node space elements. This simulates the tensile, compressive, and torsional characteristics between the mechanical joint surfaces. This modeling technique is used to investigate the dynamic properties of the bed of the PTP160PLUS Woodworking CNC Machining Center (hence referred to as the bed), construct a finite element model of the bed, and perform a modal analysis on the model. With the use of the hammer method, the bed is carried through its modal test, and the results of the test are used to validate the modeling method. On the basis of the results of the modal analysis, the weak links of the bed structure are identified, and the bed structure is adjusted using the sensitivity analysis method in order to enhance the dynamic properties of the bed.

The rest of the paper is organized as follows. In Section 2, the bed finite element modeling is discussed. In Section 3, the dynamic analysis and modal test of bed are discussed. The bed structure is optimized in Section 4. Finally, the conclusion is given in Section 5.

## 2. Bed finite element modeling

### 2.1. Modeling method of joint surface

The modeling at the joint surface is based on its dynamic characteristics, which is discretized and applied to the finite element model of the integrated composite structure. Literature [14] pointed out that stiffness, damping, and surface pressure of the joint surface show a continuous power function relationship, but the difference is not large. In the elastic range, the stiffness, damping, and surface pressure of the joint surface can be simplified to linear calculations. The finite element method is adopted to divide the joint mesh. If the mesh of the joint surface is sufficiently thin, characteristics of the joint surface will be equivalent to the joint between the joint nodes [15, 16]. Therefore, this paper uses the joint surface nodes to establish the joint surface equivalent model the joint is evenly divided into enough regular shape elements and nodes while ensuring one-to-one correspondence of nodes on the bonding surface and between each pair of bonding nodes [17]. A set of 12-degree-of-freedom space spring damping unit is adopted to simulate the property between the bonding faces. The reasons for using this unit to express the properties between binding surfaces are as follows:

1. The nodes at both ends of the cell contain 6 degrees of freedom, which is consistent with the interaction between the binding surface, so the unit can accurately express the binding surface properties.
2. The interactions between the binding surfaces are dispersed to the units between the nodes to transform the nonlinear problem into a linear problem to facilitate the numerical calculation.
3. Each unit has no spatial position relationship and quality characteristics, and will not add interference factors to the whole system.
4. The interaction relationship between the units can be realized by the boundary coordination equation and the constraint equation between the nodes.

**Fig 1:**
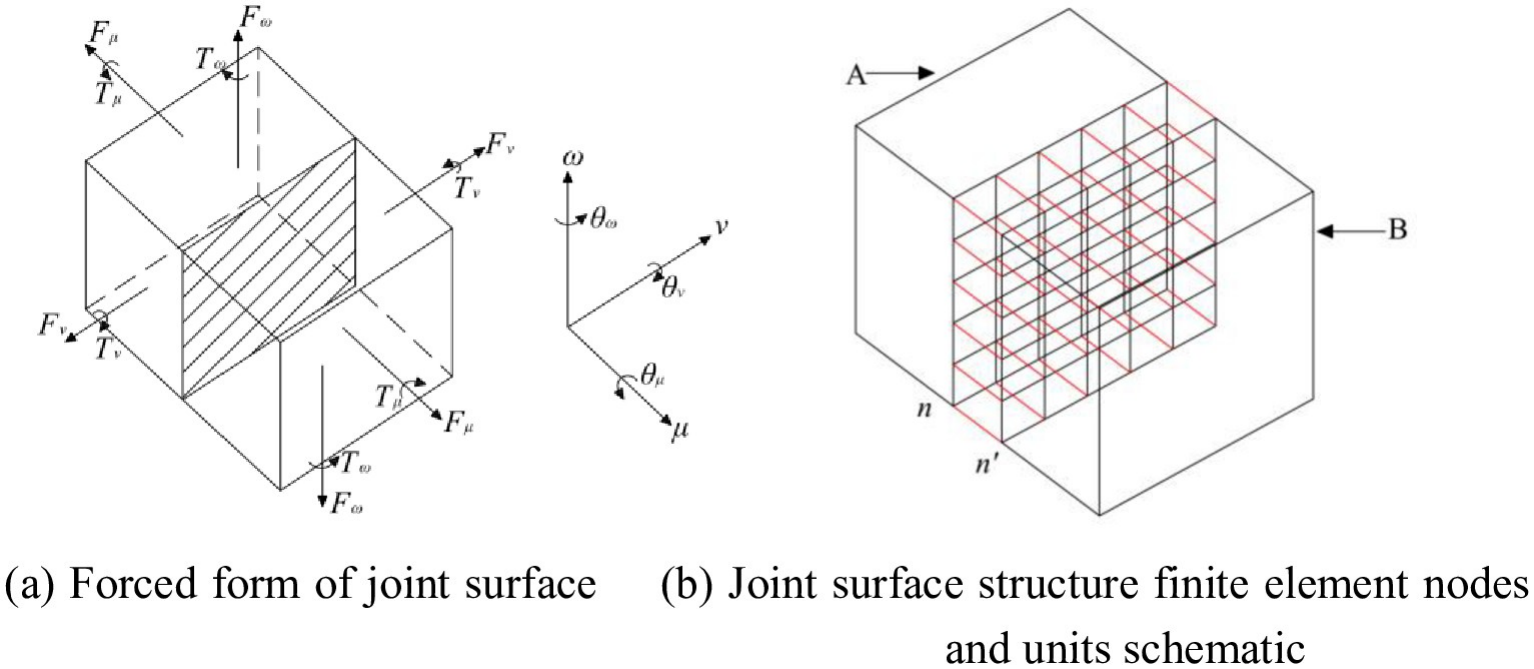
Joint surface of an actual woodworking machine structure. (a) Forced form of joint surface, (b) Joint surface structure finite element nodes and units schematic.

In the joint plane in any actual woodworking machine structure, there are generally six forms of forces at the joint surface [9] (Fig 1a). These forces include the positive force in the *μ* direction *F*_*μ*_, the shear forces *F*_*ν*_ and *F*_*ω*_ in *ν* and *ω* directions, the bending moments *T*_*ν*_ and *T*_*ω*_ around the *ν* and *ω* directions, and the torque *T*_*μ*_ around the *μ* axis.

The finite element method is adopted to evenly divide the contact joint of an actual machine tool structure (Fig 1b), and obtain n sets of joint nodes. Any joint node *i* is selected on a certain structure of the joint surface, and there will be corresponding joint node *j* on the corresponding joint surface. A joint space stiffness damping unit is similar to the beam unit established between the joint nodes, and an equation at the joint surface is obtained according to the equilibrium equation of the joint surface [18]:

$$\left\{\begin{array}{c} F_{\mu }=\sum_{i=1}^{i=n}K_{\mu i}\times \Delta \mu_{i}\\ F_{\nu }=\sum_{i=1}^{i=n}K_{\nu i}\times \Delta \nu_{i}\\ F_{\omega }=\sum_{i=1}^{i=n}K_{\omega i}\times \Delta \omega_{i}\\ T_{\mu }=\sum_{i=1}^{i=n}K_{\theta \mu i}\times \Delta \omega_{\mu i}\\ T_{\nu }=\sum_{i=1}^{i=n}K_{\theta \nu i}\times \Delta \theta_{\nu i}\\ T_{\omega }=\sum_{i=1}^{i=n}K_{\theta \omega i}\times \Delta \theta_{\omega i} \end{array}\right.$$ (1)

where, *F*_*μ*_, *F*_*ν*_, and *F*_*ω*_ are the loads in the *μ*, *ν*, and *ω* directions of the joint surface, respectively; *T*_*μ*_, *T*_*ν*_, and *T*_*ω*_ are the torque and bending moment of the joint surface in the *μ*, *ν*, and *ω* directions, respectively; *K*_*μi*_ and *K*_*θμi*_ are the normal stiffness and torsional stiffness between the joint nodes; *K*_*νi*_ and *K*_*ω*__*i*_ are the tangential stiffness between the joint nodes; *K*_*θνi*_ and *K*_*θ*__*ω*__*i*_ are the tangential torsional stiffness between the joint nodes; Δ*μ*_*i*_ is the normal deformation amount between the joint nodes; Δ*ν*_*i*_ and Δ*ω*_*i*_ are the amount of tangential deformation between the joint nodes; Δ*ω*_*μi*_, Δ*θ*_*νi*_, and Δ*θ*_*ω*__*i*_ are the torsional angular displacement between the joint nodes; and *n* is the number of joint nodes.

**Fig 2:**
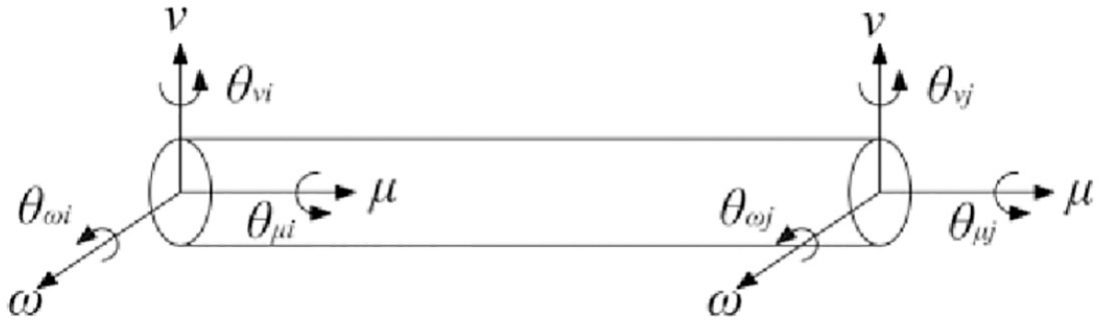
Space unit composed of joint node.

For any group of spring damping elements in space that satisfy the tension, compression, and torsion characteristics between nodes in the joint region (Fig 2), the nodes that make up the element have 6 degrees of freedom to ensure the properties of the joint region, and the number of nodes that make up the space unit of this area is no more than 2. In the process of interaction, there will be elastic or plastic deformation between nodes [19], so the joint node satisfies the deformation relationship of two nodes, namely:

$$\left[\begin{array}{l} F_{\mu i}\\ F_{\nu i}\\ F_{\omega i}\\ T_{\mu i}\\ T_{\nu i}\\ T_{\omega i}\\ F_{\mu j}\\ F_{\nu j}\\ F_{\omega j}\\ T_{\mu j}\\ T_{\nu j}\\ T_{\omega j} \end{array}\right]={\left[K\right]}_{12\times 12}\left[\begin{array}{l} \mu_{i}\\ \nu_{i}\\ \omega_{i}\\ \theta_{\mu i}\\ \theta_{\nu i}\\ \theta_{\omega i}\\ \mu_{j}\\ \nu_{j}\\ \omega_{j}\\ \theta_{\mu j}\\ \theta_{\nu j}\\ \theta_{\omega j} \end{array}\right]+{\left[C\right]}_{12\times 12}\left[\begin{array}{l} {\dot{\mu }}_{i}\\ {\dot{\nu }}_{i}\\ {\dot{\omega }}_{i}\\ {\dot{\theta }}_{\mu i}\\ {\dot{\theta }}_{\nu i}\\ {\dot{\theta }}_{\omega i}\\ {\dot{\mu }}_{j}\\ {\dot{\nu }}_{j}\\ {\dot{\omega }}_{j}\\ {\dot{\theta }}_{\mu j}\\ {\dot{\theta }}_{\nu j}\\ {\dot{\theta }}_{\omega j} \end{array}\right]$$ (2)

where, [*K*] is the stiffness matrix between the joint nodes; [*C*] is the damping matrix between the joint nodes; *F*_*μi*_, *F*_*νi*_, and *F*_*ω*__*i*_ are the loads in the *μ*, *ν*, *ω* directions of the node *i*, respectively; *F*_*μj*_, *F*_*νj*_, and *F*_*ω*__*j*_ are the loads in the *μ*, *ν*, and *ω* directions of the node *j*, respectively; *μ*_*i*_, *ν*_*i*_, and *ω*_*i*_ are the displacements of the joint node *i* in the *μ*, *ν*, and *ω* directions, respectively; *μ*_*j*_,*ν*_*j*_, and *ω*_*j*_ are the displacements of the joint node *j* at *μ*, *ν*, and *ω* directions, respectively; *T*_*μi*_, *T*_*νi*_, and *T*_*ω*__*i*_ are the bending moments and torques in the *μ*, *ν*, and *ω* directions of the joint node *i*, respectively; *T*_*μj*_, *T*_*νj*_, and *T*_*ω*__*j*_ are the bending moments and torques in the *μ*, *ν*, and *ω* directions of the joint node *j*, respectively; *θ*_*μi*_, *θ*_*νi*_, and *θ*_*ω*__*i*_ are torsional angular displacements in the *μ*, *ν*, and *ω* directions received by the joint node *i*, respectively; and *θ*_*μj*_, *θ*_*νj*_, and *θ*_*ω*__*j*_ are torsional angular displacements in the *μ*, *ν*, and *ω* directions received by the joint node *j*, respectively.

When there is a joint structure modeling, the nodes of the joints have correspondence. Therefore, the stiffness matrix [*K*] and the damping matrix [*C*] of the joint unit are symmetric matrices.

$$K=\left[\begin{array}{ccccccc} K_{11} & \cdots & K_{1i} & \cdots & K_{1j} & \cdots & K_{1n}\\ \vdots & & \vdots & & \vdots & & \vdots \\ K_{i1} & \cdots & K_{ii} & \cdots & K_{ij} & \cdots & K_{in}\\ \vdots & & \vdots & & \vdots & & \vdots \\ K_{j1} & \cdots & K_{ji} & \cdots & K_{jj} & \cdots & K_{jn}\\ \vdots & & \vdots & & \vdots & & \vdots \\ K_{n1} & \cdots & K_{ni} & \cdots & K_{nj} & \cdots & K_{nn} \end{array}\right]C=\left[\begin{array}{ccccccc} C_{11} & \cdots & C_{1i} & \cdots & C_{1j} & \cdots & C_{1n}\\ \vdots & & \vdots & & \vdots & & \vdots \\ C_{i1} & \cdots & C_{ii} & \cdots & C_{ij} & \cdots & C_{in}\\ \vdots & & \vdots & & \vdots & & \vdots \\ C_{j1} & \cdots & C_{ji} & \cdots & C_{jj} & \cdots & C_{jn}\\ \vdots & & \vdots & & \vdots & & \vdots \\ C_{n1} & \cdots & C_{ni} & \cdots & C_{nj} & \cdots & C_{nn} \end{array}\right]$$

where, *K*_*ij*_ = *K*_*ji*_ *i* ≠ *j*; *C*_*ij*_ = *C*_*ji*_ *i* ≠ *j*; and *n* = 12.

There is no mass at the joint surface. According to the principle of continuous system discretization, the multi-degree-of-freedom system at the joint surface can be expressed as follows:

$$K\left\{q\right\}+C\left\{\dot{q}\right\}=\left\{F\right\}$$ (3)

where, {*q*} = [*μ ν* *ω*], *K* and *C* are the stiffness matrix and damping matrix of the joint, respectively, and *F* is the external load of the joint node.

The positive direction of the vector is specified, and relationships between the stiffness matrix of each joint unit, the damping matrix, and the stiffness damping of the joint region are obtained from Eqs (2) and (3), respectively; and the model at the joint surface is established according to Eq (1). For any substructure: the finite element model can be established based on the dynamic equation (Eq (4)) of the continuous system of the structure itself. The model at the joint surface is coupled with the model of each substructure through common nodes and units to establish a machine tool finite element model with the characteristics of the joint surface.

$$M_{r}\left\{\ddot{q}\right\}+C_{r}\left\{\dot{q}\right\}+K_{r}\left\{q_{r}\right\}=\left\{F_{r}\right\}$$ (4)

where, {*q*_*r*_} = [*μ*_*r*_ *ν*_*r*_ *ω*_*r*_]; *M*_*r*_ is the mass matrix of the substructure; *K*_*r*_ is the stiffness matrix of the substructure; *C*_*r*_ is the damping matrix of the substructure; and *F*_*r*_ is the external load of the node at the substructure.

In addition to ensuring uniform joint surface of the bed and the correspondence between the joints, the accurate rigidity and damping of the joint surface of the machine tool can’t be ignored in the overall modeling process of the bed. At present, the stiffness and damping of the joint are mainly obtained by identifying the test data and the model. The identified joint stiffness and damping are substituted into the stiffness matrix and damping matrix of Eq (2) to establish the joint unit model. The subunits are associated with each substructure model to establish a bed model containing the dynamic characteristics of the joint surface.

### 2.2. Establishment of finite element model of woodworking CNC machining center bed

#### 2.2.1 Bed finite element analysis process

**Fig 3:**
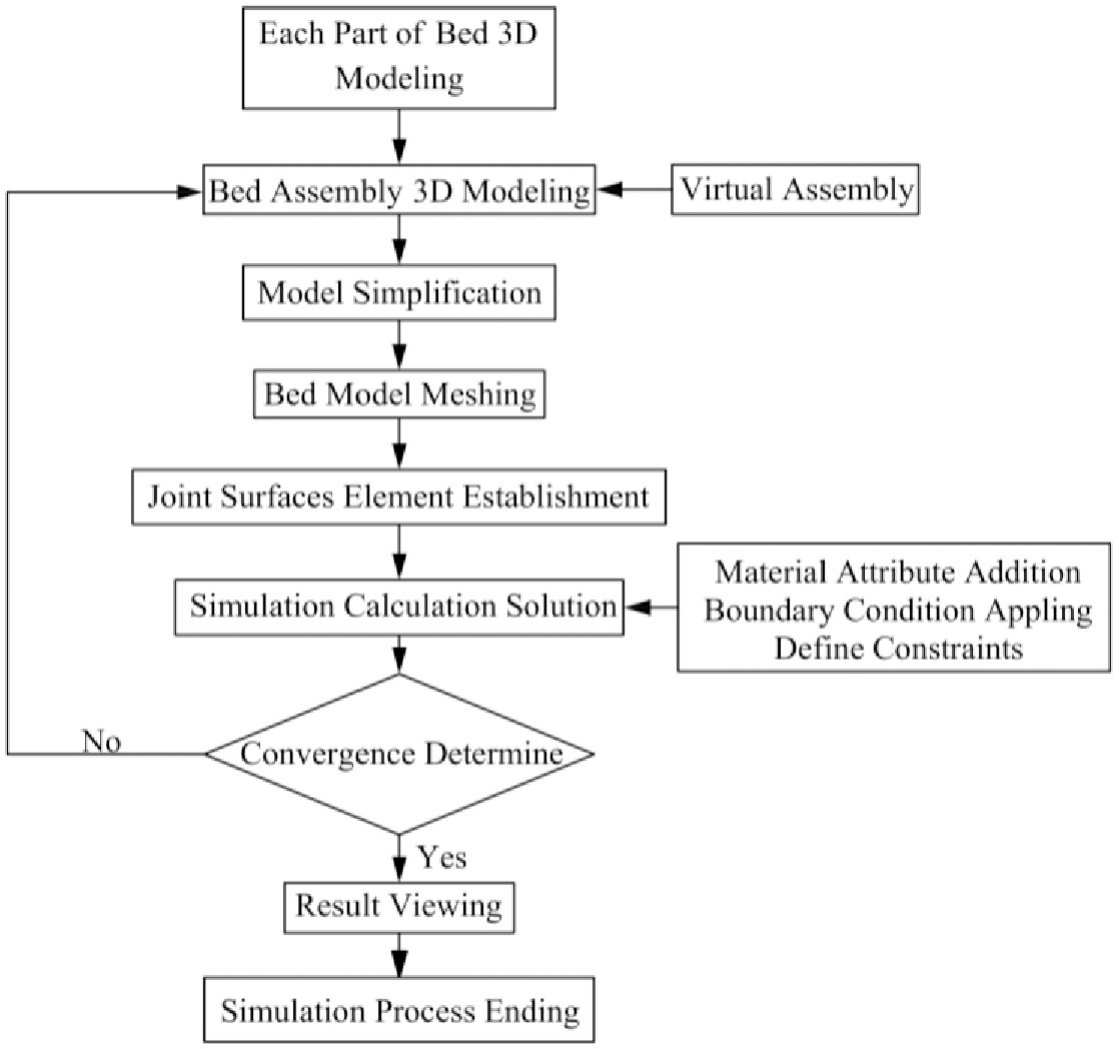
Bed finite element analysis process.

Typically, the finite element analysis process consists of three phases. The first part is the model’s pre-processing. The structure to be analyzed’s geometric model, or 3D modeling, is established first. In this work, SolidWorks is used to create a 3D model of the bed, which is then meshes using a mesh division program. The second step is to specify the solution condition, which is the crux of finite element analysis and includes model selection, material creation, setting of constraint and boundary conditions, etc. In this study, HyperMesh is used to divide the mesh, Ansys is used to set the solution condition, and finite element solution is carried. The final part is post-processing. The post-processor can perform graphical display and statistics on the data obtained after solving in order to examine the calculation results, build and display the system displacement cloud map, and so forth. Fig 3 depicts the process of bed finite element analysis.

#### 2.2.2. Bed 3D modeling

**Fig 4:**
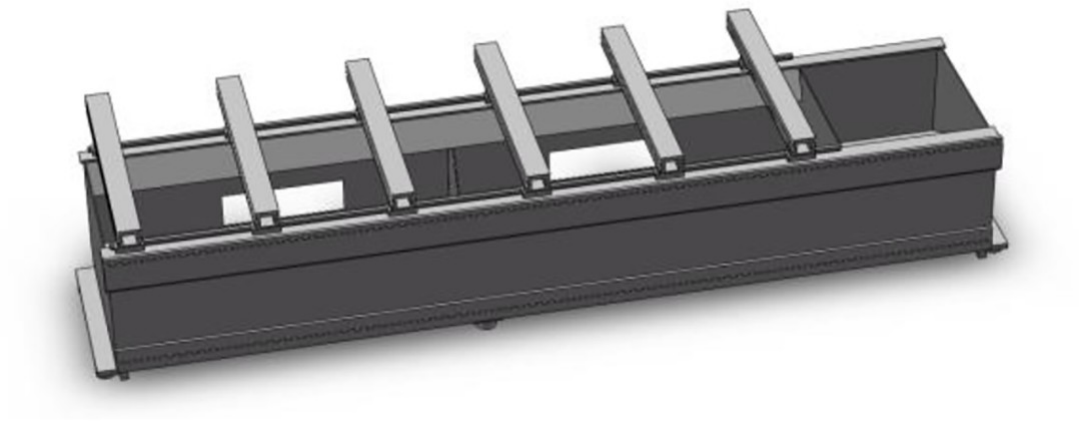
Bed simplified 3D solid model.

When the finite element model is established, the 3D model of the bed is firstly established based on its actual structure. If the fine features such as rounded corners, chamfers, bolt holes, and small bosses in the bed are all reflected in the model, the number of cell grids is huge, the calculation accuracy is degraded, and the error of the simulation analysis is increased, greatly affecting the simulation calculation result [20]. Therefore, the bed structure can be simplified to improve the accuracy and accelerate the speed of the bed meshing and finite element analysis. Fig 4 is a simplified 3D solid model of the bed.

#### 2.2.3. Bed model meshing

In this paper, Hypermesh software is employed to mesh the bed model, and enough units and nodes with regular shapes are divided at each joint surface to ensure one-to-one correspondence of the nodes on the joint surface. In this way, it can establish the equivalent dynamic model of the joint surface through these nodes, and set the material properties of the various parts of the bed in the Hypermesh software, the material properties of each part of the bed are shown in Table 1, 458,231 units and 151,046 nodes are divided into the bed.

**Table 1:** Material properties of each part of bed.

Structure	Material	Elasticity modulus (GPa)	Density (kg/m^3^)	Poisson’s ratio (N/m)
Workbench	AlMg0.7Si	69	2690	0.33
Slider	GCr15	219	7800	0.3
Bed	Q235	200	7860	0.288
Support	HT300	143	7300	0.27
Guide	GCr15	219	7800	0.3

#### 2.2.4. Joint surface modeling

The main joint surfaces in the bed are between the bed and the support, between the bed and the slider, and between the support and the slider. There is a relationship between the stiffness at the joint surface, the damping, and the surface pressure of the joint surface. In the continuous power function relationship, according to the influence cone theory [21, 22], the main concentration range of the surface pressure generated by the bolt pre-tightening force is the cone area near the circular gasket, and the angle of action is generally conservatively selected *a* = 30°. Since the bolts on the bed are evenly distributed and dense enough, the stiffness and damping at the joint surface are more evenly distributed. According to Yoshimura’s unit area integral method [23], the equivalent stiffness of the fixed joint surface can be calculated according to Eqs (5) and (6).

$$K_{1}=\iint k_{1}(p)\text{d}y\text{d}z$$ (5)

$$K_{2}=\iint k_{2}(p)\text{d}y\text{d}z$$ (6)

where, *K*_1_ and *K*_2_ are the normal stiffness and tangential stiffness of the joint surface, respectively; and *k*_1_(*p*) and *k*_2_(*p*) are the normal stiffness and tangential stiffness per unit area, respectively.

The base of the machine bed and the ground are connected to the ground by bolts, and the simulation of the boundary conditions is defined by the fixed joint surface. The data per unit area of the fixed joint surface used in this paper is the basic data of the test. According to the bonding conditions of the joint surface (material, surface pressure, joint surface medium, roughness, etc.), the characteristic parameters of the unit area joint surface are selected and substituted into the Eqs (5) and (6) to calculate the equivalent characteristic of the actual joint surface (Table 2). The surface pressure refers to the positive pressure per unit area of the joint surface, which is calculated by the combination of bolt preload, component gravity, and cutting force. Similarly, the equivalent of damping can be completed.

**Table 2:** Joint stiffness and damping characteristics.

Structure 1	Structure 2	Normal stiffness (N/m)	Tangential stiffness (N/m)	Normal damping (N/m)	Tangential damping (N/m)
Workbench	Slider	5.87×10^8^	2.21×10^8^	1.45×10^2^	3.73×10^3^
Slider	Guide	6.28×10^8^	5.87×10^8^	1.80×10^2^	2.90×10^3^
Bad	Support	5.03×10^9^	6.10×10^8^	3.35×10^2^	4.10×10^3^
Support	Earth	6.27×10^9^	5.33×10^8^	2.17×10^2^	3.57×10^3^

**Fig 5:**
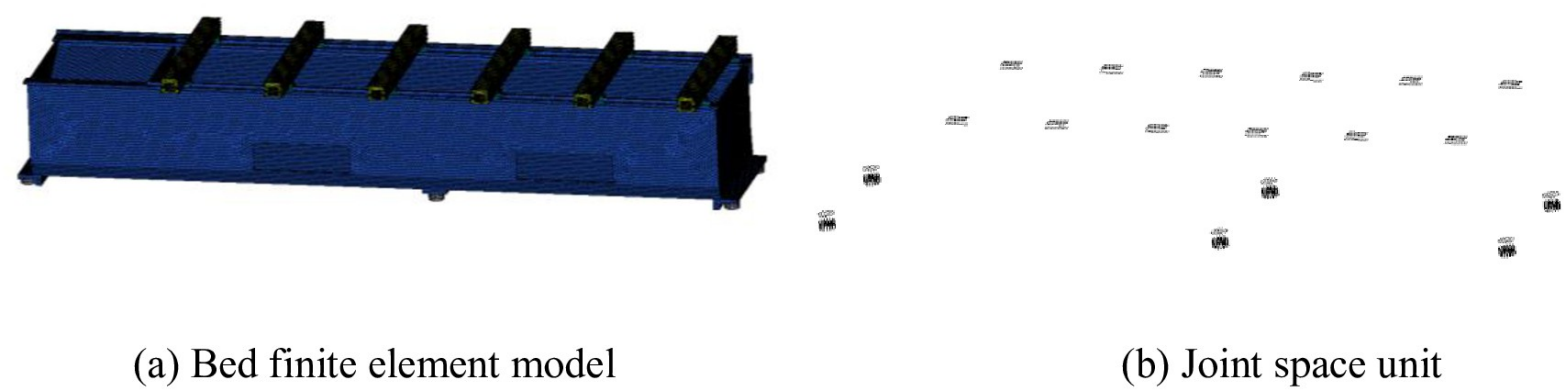
Bed structure finite element model and joint space unit. (a) Bed finite element model, (b) Joint space unit.

In this paper, the matrix unit Matrix27 is undertaken as the connection unit to integrate the characteristics of the joint surface. The Matrix27 unit is created on the joint surface generated during meshing. According to the parallel principle of the spring and the number of joint units [24], the characteristics of the joint surface are discretized to each unit, and the normal and tangential stiffnesses of the single unit on different joints are obtained, the command stream that used to add Matrix27 unit is shown in S1 Text. The Matrix27 element rigid and damp real constants are set to give the joint surface stiffness and damping property. The bed finite element model and joint space unit diagrams are shown in Fig 5.

## 3. Dynamic analysis and modal test of bed in woodworking CNC machining center

### 3.1. Finite element modal analysis

The finite element modal of the bed-limited element model is analyzed by ANSYS. According to the actual working conditions of the woodworking CNC machining center, the modal is analyzed for the bed model considering the dynamic characteristics of the joint surface and the machine bed model with rigid connection of the joint surface. The block Lanczos method (Block Lanczos) is employed to extract the natural frequencies and modes [25]. Generally, the actual working speed and the frequency of the woodworking CNC machining center are5,000 ~ 15,000 r/min and 83 Hz ~ 250 Hz, respectively. Therefore, the first 6^th^ order modal data are taken according to the modal calculation result, and its natural frequency and mode shape description are shown in Table 3. *f* is the frequency of each step of the bed obtained by the modal test; and *f*_1_ and *f*_2_ refer to the analysis frequency obtained by the finite element simulation of the bed with and without the characteristics of the joint surface, respectively.

**Table 3:** Comparison of test frequency and simulation analysis frequency.

Order	*f*/Hz	*f*_1_/Hz	Relative error (%)	*f*_2_/Hz	Mode description
1	56.32	67.33	19.55	74.81	The left side of the front wall of the bed is slightly convex, and the left slider slides in the Y direction.
2	93.09	101.88	9.44	110.46	Overall sliding deformation along the X direction
3	139.43	146.75	5.25	157.87	The left side of the front wall of the bed is convex
4	170.18	179.75	5.62	188.30	The front side of the front wall of the bed is convex, and the center of the right side of the table is concave.
5	210.91	213.37	2.13	226.51	The right side of the bed swings to the left and right, and the front wall of the bed is torsionally vibrated along the middle surface.
6	252.77	255.49	1.08	263.29	The front wall of the bed and the table are torsional along the middle surface

### 3.2. Bed mode test

#### 3.2.1. Test equipment

**Fig 6:**
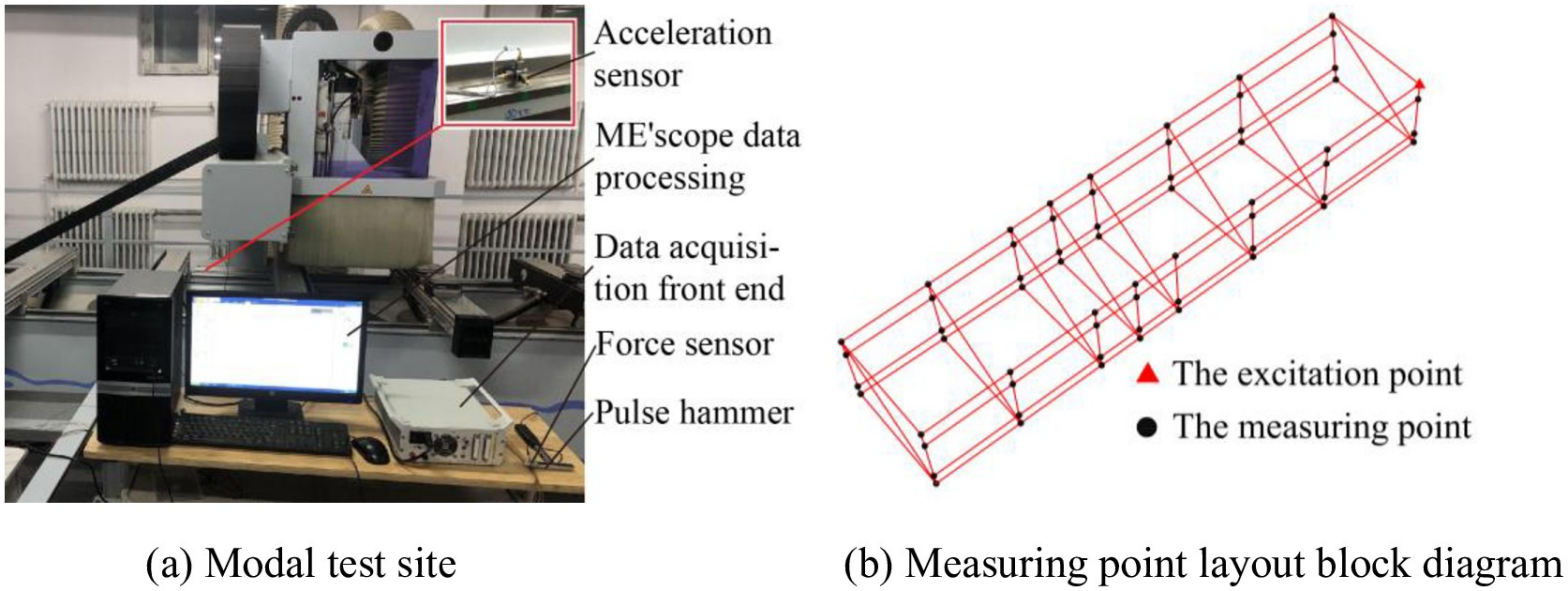
Modal test site and test point layout. (a) Modal test site, (b) Measuring point layout block diagram.

To compare with the analysis frequency and vibration mode obtained by the limit element simulation, the validity of the characteristics of the joint surface modeling method is verified, and the bed structure is tested by the hammer method [26]. The test equipment mainly includes the pulse hammer, the acceleration sensor, the BK multi-channel data acquisition front end, and the ME’scope data processing system. The test modal test site is shown in Fig 6(a).

#### 3.2.2. Test method and test point

The single-point excitation multi-point response is adopted in the test. According to the result of finite element analysis, the point in the upper right corner of the workbench is selected as the excitation point. Based on the test point, structure of the whole system is reflected as much as possible without missing the principle of the mode [27]. 63 measuring points are arranged on the body. Fig 6(b) is a block diagram of the measuring point arrangement on the bed in the test. The intersection of each line is the position where the measuring point is.

### 3.3. Comparison of test and simulation modes and data

**Fig 7:**
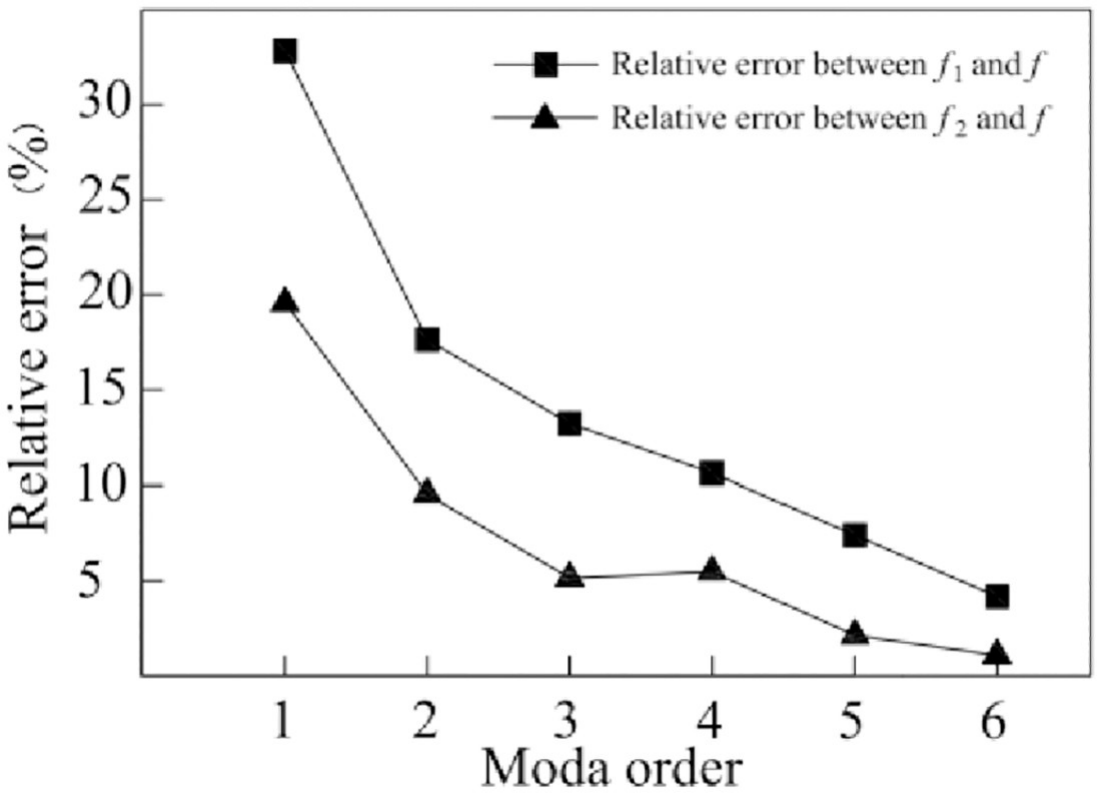
Relative error between simulation result and test result.

**Fig 8:**
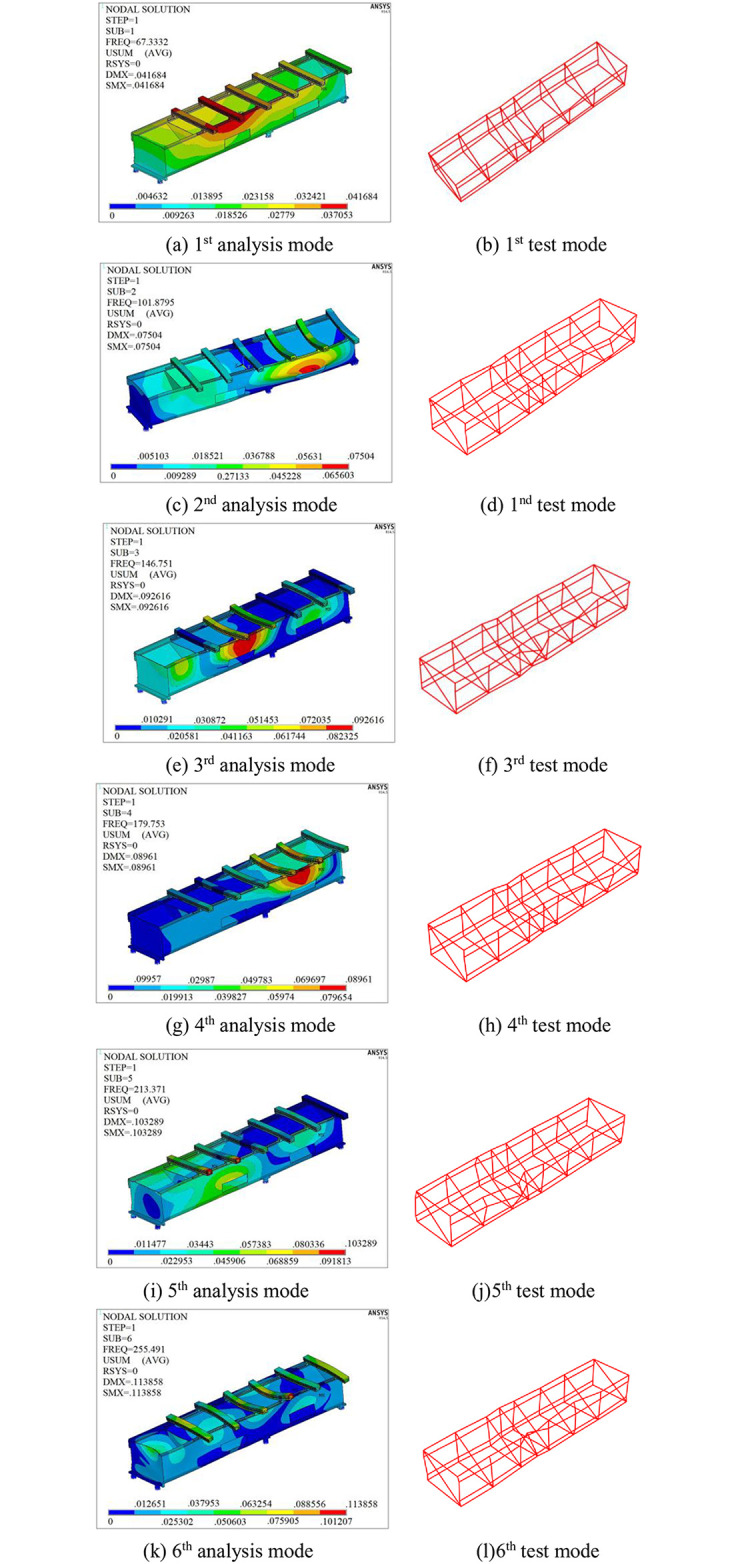
Comparison of modal modes between finite element modes and experimental modes. (a) 1^st^ analysis mode, (b) 1^st^ test mode, (c) 2^nd^ analysis mode, (d) 1^nd^ test mode, (e) 3^rd^ analysis mode, (f) 3^rd^ test mode, (g) 4^th^ analysis mode, (h) 4^th^ test mode, (i) 5^th^ analysis mode, (j)5^th^ test mode, (k) 6^th^ analysis mode, (l)6^th^ test mode.

Frequency in the modal test is shown by *f* in Table 3, the error between the simulation and test result is shown in Fig 7, and the comparison between the mode vibration obtained by the modal test and that from the simulation analysis is shown in Fig 8. In Fig 7, the comparison of the simulation analysis and frequency in the modal test shows that resulting error of the bed model without characteristics of the joint surface is 13.28 ~ 3.08% larger than that of the bed model with, and latter is closer to reality. The first-order analysis frequency of the bed model with the characteristics of the joint surface shows a larger error of 19.55% compared with the modal test result, which may be because that the first-order vibration deformation mainly occurs on the slider structure. As the slider structure is too small, the test point can’t be directly arranged on the slider structure during the modal test, so a nearby point is adopted instead. The error between analysis frequency of other order and test frequency is 1.08% ~ 9.44%. According to Fig 8, the simulation analysis and the test order mode are almost the same. The comparison of conditions between the simulation analysis and the modal test suggests that the data error between the simulation and the test result may be caused by following two aspects:

1. The basic data of the joint surface in the modeling process is measured according to the test, and there is an error.
2. In the modal test, the energy input to the system by the hammer stroke is limited, so the accuracy of the signal received by the sensor decreases.

Despite of the numerical error between the simulation result and the test result, the data comparison suggests that in the bed modeling process, the bed model with the characteristics of the joint surface is closer to the modal test result, with a reasonable error. The above result shows that the bed model with the characteristics of the joint surface can better reflect the dynamic performance of the bed. This paper using the equivalent modeling method of the joint surface is proved effective, accurately reflecting the characteristics of the bed joint surface. In addition, the bed model established based on characteristics of the joint surface in this paper is proved reasonable and can be popularized and applied to other woodworking machinery for analysis of dynamic performance.

According to the analysis and test vibration mode result, vibration deformation of the bed mainly occurs on the workbench. The workbench can greatly affect the machining precision of the woodworking CNC machining center. It should be considered in design optimization and increase the thickness or ribs inside the workbench. The vibration mode of the bed suggests that the front wall shows insufficient bending and torsion resistance, and the torsion and flexural rigidity can be improved by appropriately increasing the thickness of the sheet metal or arranging the ribs inside the bed.

## 4. Optimization of bed structure for the woodworking CNC machining center

According to the simulation analysis and experimental modal result, vibrations of the bed and the front wall are more obvious. The modal analysis is performed to optimize the bed structure, and the result of the finite element analysis of the bed model before and after optimization are compared to verify the optimization accuracy.

### 4.1. Establishment of the bed optimization model

**Fig 9:**
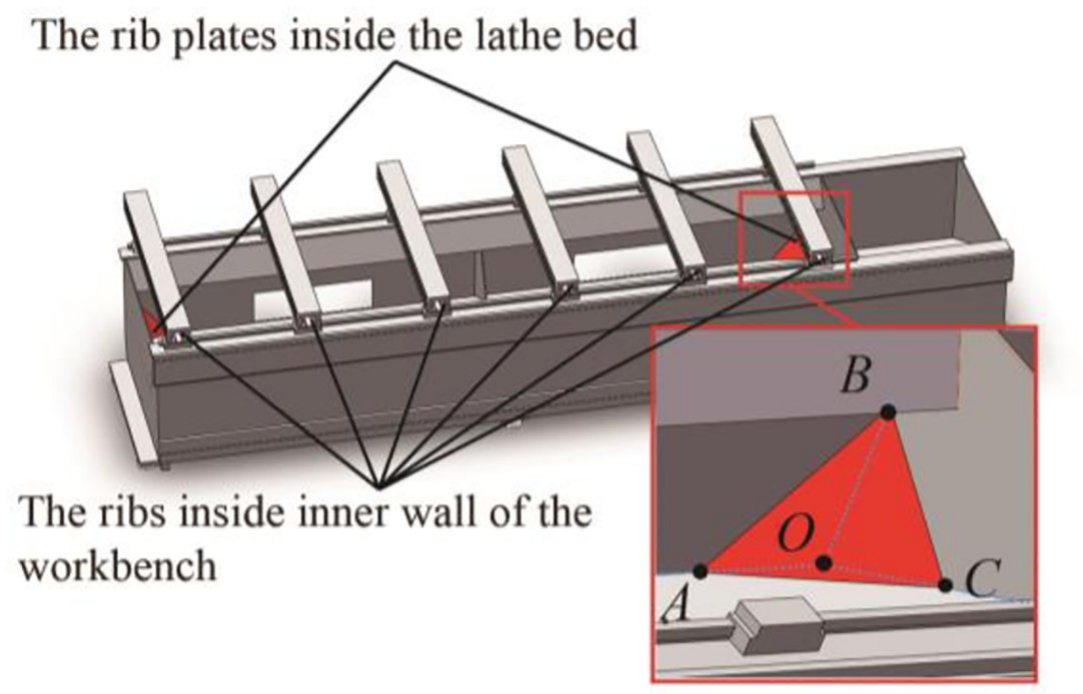
Optimized bed 3D solid model model.

According to the simulation analysis and experimental modal result, the weak part of the bed is optimized in the SolidWorks software. Two ribs are added to the inner wall of each workbench, two rib plates are added to the left and right sides on the front metal plate inside the bed, respectively. The optimized bed 3D solid model model is shown in Fig 9.

In this optimization, considering the computation amount, only seven parameters are chosen as the input quantity: the distances *L*_1_, *L*_2_, *L*_3_ between the end points A, B, C of the left side rib plate and the O intersection point of the bed’s sheet metal, the thickness *D* of the rib plate, and the width *W* and height *H* of the rib. On the body mass, natural frequency, and relative displacement, the effects of these seven parameters are studied. Table 4 displays the range of values for each parameter.

**Table 4:** Optimized parameters and their value ranges.

Parameter variable	Description	Variation range/mm
*L*_1_	The distance between the end point A of the rib plate and the intersection point O of the sheet metal of the bed	100~350
*L*_2_	The distance between the end point B of the rib plate and the intersection point O of the sheet metal of the bed	100~320
*L*_3_	The distance between the end point C of the rib plate and the intersection point O of the sheet metal of the bed	100~350
*D*	The thickness of the rib plate	4~10
*W*	The width of the rib	4~12
*H*	The height of the rib	4~12

**Fig 10:**
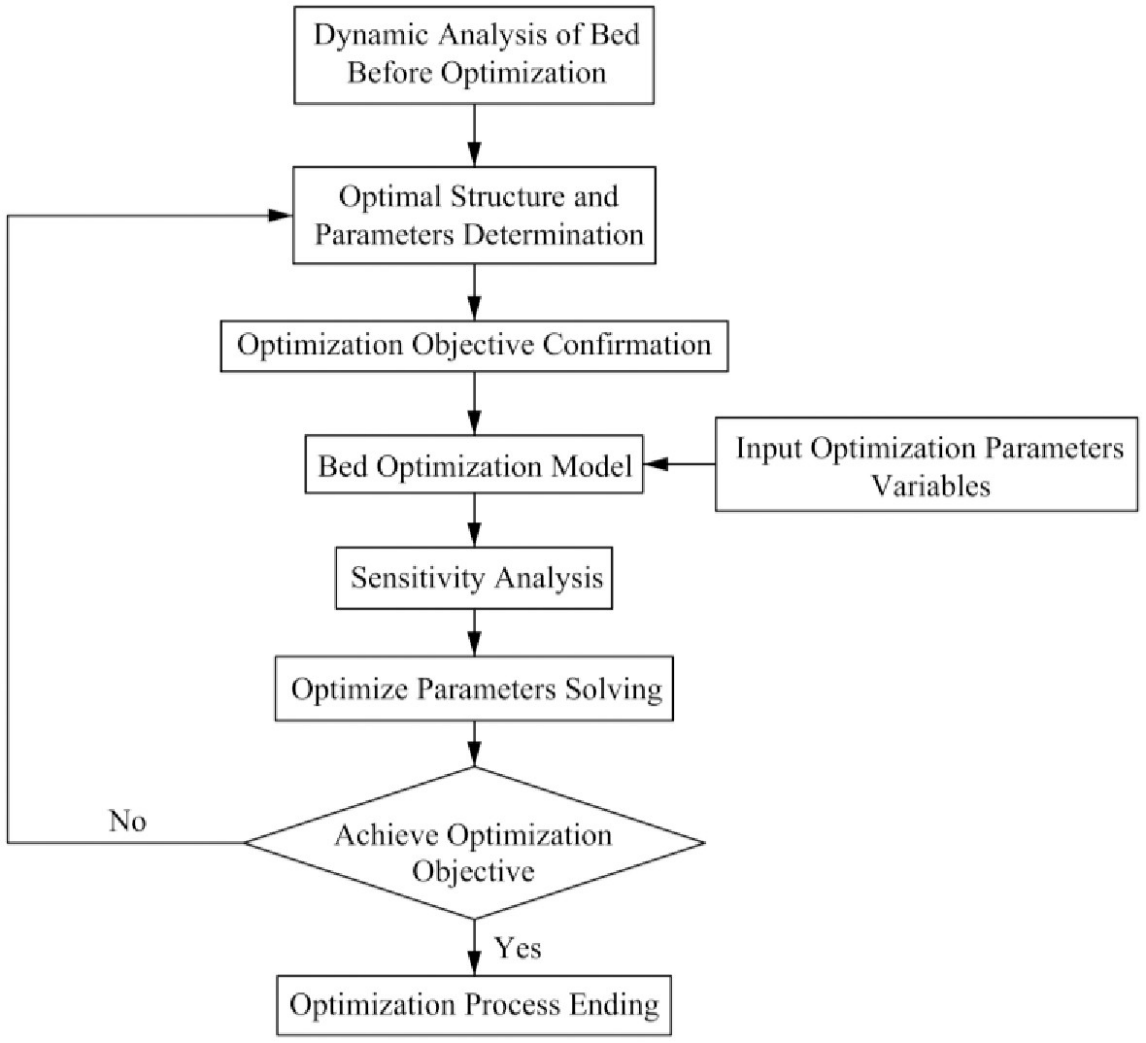
Flow chart of bed structure optimization.

The mass of the bed, the maximum relative displacement, and the first six natural frequencies are set as the objective functions for the structure optimization of the bed, and an optimization model is established. Fig 10 depicts the bed structure optimization process.

### 4.2. Optimization of bed structure

**Fig 11:**
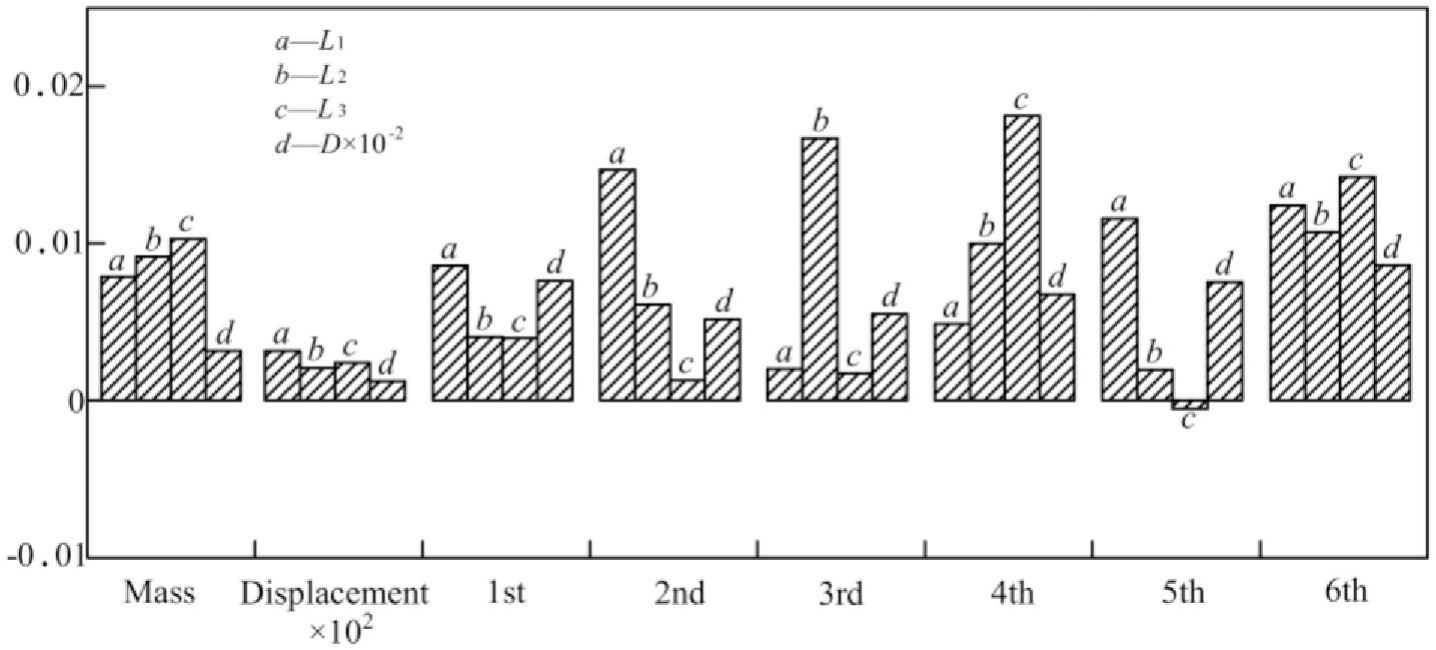
Sensitivity analysis chart of *L*_1_, *L*_2_, *L*_3_ and *D* to each performance.

The sensitivity analysis method, which analyzes the sensitivity of structural performance function changes to structural design parameter changes, is used to optimize the bed structure. Using the sensitivity analysis function of the finite element analysis program ANSYS Workbench, the optimal parameter size can be easily identified. Since the reb on the workbench and the reb plate on the bed body act on distinct components, they can be taken into account individually during the optimization process to establish the optimal size. Fig 11 displays the sensitivity of the four reb plate parameters *L*_1_, *L*_2_, *L*_3_ and *D* to the first six natural frequencies, as well as the maximum relative displacement and bed mass.

The software ANSYS Workbench is utilized to optimize the bed’s structure, with the maximum sixth-order natural frequency, the least maximum relative displacement, and the maximum bed mass serving as optimization objectives. The solution is calculated using the MOGA genetic algorithm, and after multiple iterations, the optimization feasibility solution set is achieved, the solution set is listed in S1 Data. After thoroughly taking into account the four optimization objectives of the bed, three groups of relative optimal solutions Candidate A, Candidate B, and Candidate C are generated, as shown in Table 5.

**Table 5:** Structural optimization for relatively optimal solutions.

		Candidate A	Candidate B	Candidate C
*L*_1_/mm		308.17	300.40	277.08
*L*_2_/mm		297.26	317.29	304.22
*L*_3_/mm		237.43	269.39	220.10
*D*/mm		7.02	6.87	7.44
Mass/kg		1923.70	1925.81	1917.99
Displacement/mm		0.103	0.105	0.103
Mode/Hz	1st	71.95	69.21	69.13
	2st	104.42	103.11	102.79
	3st	147.91	148.05	147.75
	4st	181.57	182.03	182.04
	5st	223.36	220.37	218.66
	6st	272.02	267.84	275.94

The optimal choice for the best bed structure optimization solution is Candidate A following comparison analysis. The optimal solutions for the width *W* and height *H* of inner wall ribs in the workbench, using the same optimization solution process, are 7.94 mm and 10.11 mm, respectively. The parameters of each dimension are correctly rounded for the convenience of the real product design and manufacture, and the results are displayed in Table 6. The optimized structural parameters are taken as the structural dimensions of all the rib plates and ribs of the bed, in order to establish the optimized finite element model of the bed, and the model is entered into ANSYS to do the finite element analysis.

**Table 6:** Roundness results of optimization variables.

Dimension parameter	Rounding result/mm	Actual value/mm
*L*_1_	308	308.17
*L*_2_	297	297.26
*L*_3_	237	237.43
*D*	7	7.02
*W*	8	7.94
*H*	10	10.11

### 4.3. Comparison of finite element analysis result before and after bed optimization

According to the bed optimization scheme, the reinforcing ribs are added to the inner wall of the workbench and two ribs are added to the inner wall of the bed near the front wall. Therefore, the total mass of the woodworking CNC machining center bed is only increased by 0.42%, and the bed structure changes not greatly before and after optimization. Comparison between the result of the meta-analysis and the natural frequency before the optimization is shown in Table 7.

**Table 7:** Comparison of natural frequency before and after bed optimization.

Order	Before optimization/Hz	Optimized/ Hz	Optimized displacement/mm	Optimized relative displacement/mm
1	67.33	74.95	0.042	0.040
2	101.88	104.49	0.075	0.067
3	146.75	151.71	0.093	0.075
4	179.75	184.33	0.090	0.082
5	213.37	229.60	0.103	0.083
6	255.49	279.02	0.114	0.099

Comparison on the result of finite element analysis before and after optimization of the bed structure reveals that the natural frequency of the optimized bed structure is increased by 2.55% ~ 11.3%, and the corresponding relative displacement of each step is reduced with the maximum reduction value of 19.4%. It indicates that the optimized bed model shows increased rigidity, reduced vibration deformation, and greatly improved dynamic performance, achieving the design optimization.

## 5. Conclusion

1. The dynamic characteristics analysis of woodworking machine tools was conducted using a method based on the finite element method that took into analysis the dynamic characteristics of the joint surface. The equivalent model of the joint surface was established by directly simulating the tensile, compressive, and torsional characteristics between mechanical joint surfaces utilizing the nodes of the joint surface. Based on this method, the parameters for the joint surface were established in the finite element model of the bed of the woodworking CNC machining center.
2. The modal analysis results of the bed model with joint surface parameters were more compatible with the test results when compared to the simulation results of the model without joint surface parameters, combined with the modal test data, and the overall error was within 19.55%. It demonstrates that examining the dynamic characteristics of the joint surface is crucial for analyzing the dynamic characteristics of the bed, and that the modeling method of the joint surface presented in this study is effective.
3. The weak points of the bed structure were improved and optimized in accordance with the analysis of each mode form. The bed optimization model was established using the sensitivity analysis method, and the optimal size of the optimization parameters was determined. The natural frequency of the bed after optimization was enhanced by 2.55%-11.3% in comparison to the results of the simulation analysis before and after optimization, and the maximum related relative displacement was decreased by 19.4%. The dynamic performance of the bed was enhanced, and the intended optimization was accomplished.

## Supporting information

S1 Text Command stream that used to add Matrix27 unit. (TXT)

S1 Data Optimization feasibility solution set. (XLSX)
